# The role of induction chemotherapy followed by surgery in unresectable stage IVb laryngeal and hypopharyngeal cancers: a case series

**DOI:** 10.1186/s40463-018-0310-y

**Published:** 2018-10-16

**Authors:** Pichit Sittitrai, Donyarat Reunmarkkaew, Saisaward Chaiyasate

**Affiliations:** Department of Otolaryngology, Chiang Mai University, Chiang Mai, 50200 Thailand

**Keywords:** Larynx, Hypopharynx, Chemotherapy, Unresectable tumor

## Abstract

**Background:**

The purpose of this study was to evaluate the benefit of induction chemotherapy followed by surgery in locally advanced unresectable stage IVb laryngeal and hypopharyngeal squamous cell carcinoma (LHSCC).

**Methods:**

Data of patients with stage IVb LHSCC who received induction chemotherapy for the purpose of tumor resection between January 2007 and January 2016 were retrospectively collected. Definitive surgery with postoperative adjuvant therapy was performed in patients whose tumors became resectable (resectable group). Chemoradiotherapy, radiotherapy, or supportive care was considered in patients whose tumors remained unresectable (unresectable group).

**Results:**

Thirty-two patients were identified; the tumor resectability rate after induction chemotherapy was approximately 56%. The median overall survival (OS) rates of the resectable and unresectable groups were 20.0 months (range, 16.0–35.5 months) and 9.5 months (range, 6.0–15.0 months), respectively (*p* = 0.008). The estimated 2-year OS rates of the resectable and unresectable groups were 59.5% (95% confidence interval [CI], 33.2–78.3%) and 10.7% (95% CI, 1.1–35.4%), respectively (*p* = 0.008). The estimated 2-year disease-free survival (DFS) rates of the resectable and unresectable groups were 53.5% (95% CI, 27.9–73.6%), and 14.3% (95% CI, 2.3–36.6%), respectively (*p* = 0.009). On multivariate analysis, factors positively impacting OS and DFS in all patients were surgical resection, a laryngeal primary site, and induction chemotherapy with docetaxel, cisplatin, and fluorouracil.

**Conclusions:**

In advanced unresectable stage IVb LHSCC patients, surgical resection following induction chemotherapy appears to improve survival outcomes.

## Background

Head and neck squamous cell carcinoma (HNSCC) accounts for approximately 6% of all cancers worldwide; most patients present with locally advanced diseases [1–3]. The standard treatment for advanced resectable HNSCC is surgery followed by radiotherapy or a combination of chemotherapy and radiotherapy [1, 3, 4]. In more advanced unresectable tumors, radiotherapy was considered the conventional treatment [5]. However, with these modalities’ limited responses and low survival rates, alternative approaches including altered fractionation radiotherapy, combined radiotherapy and chemotherapy, and combined radiotherapy and targeted therapy were devised [5–7]. Meta-analyses and clinical trials have previously demonstrated the superiority of combined radiotherapy and chemotherapy over radiation therapy alone in advanced unresectable head and neck cancer patients; however, the survival advantage remained inadequate [6–9]. For advanced unresectable laryngeal and hypopharyngeal squamous cell carcinoma (LHSCC), multimodality treatment has also been introduced, with induction chemotherapy administered before definitive local therapy as the most promising option [1, 4, 10]. The use of induction chemotherapy to reduce tumor size and improve surgical resectability has been investigated in previous studies; however, almost all patients had oral cavity cancers, and the criteria for unresectability remain very heterogeneous [11–13].

Although the criteria for unresectability are widely debated, stage IVb HNSCC, as defined by the American Joint Committee on Cancer (AJCC) Staging Manual (7th edition), is the clearest and most accepted cutoff for resectability [14]. The purpose of this study was to evaluate the benefit of induction chemotherapy that achieved adequate tumor shrinkage followed by surgery in patients with locally advanced unresectable stage IVb laryngeal and hypopharyngeal squamous cell carcinoma.

## Methods

We conducted a retrospective study of patients with unresectable LHSCC who underwent induction chemotherapy to render tumors resectable at the Department of Otolaryngology, Faculty of Medicine, Chiang Mai University between January 2007 and January 2016. The patients were evaluated with clinical examination and imaging studies (computed tomography and/or magnetic resonance imaging); primary tumors and/or cervical lymph nodes were initially considered unresectable if they had 1) prevertebral fascia invasion, 2) carotid artery encasement of more than 270 degrees, or 3) mediastinal structure involvement. Patients who had distant metastasis, Eastern Cooperative Oncology Group performance status ≥2, or had not completed all 3 cycles of induction chemotherapy were excluded.

The induction chemotherapy regimen was as follows: 1) cisplatin 100 mg/m^2^ on day 1, and 5-fluorouracil (5-FU) 1000 mg/m^2^/d from days 1–4 (PF regimen), 2) carboplatin at an area under the curve of 5 on day 1 and paclitaxel 175 mg/m^2^ on day 1 (CP regimen), and 3) docetaxel 75 mg/m^2^ on day 1, cisplatin 75 mg/m^2^ on day 1, and 5-FU 750 mg/m^2^/d from day 1 to 4 (TPF regimen). The choice of the regimen was decided based on the patient’s performance status, creatinine clearance, and financial constraints. Induction chemotherapy was administered in 3 cycles every 3 weeks; 2–3 weeks after completing the third cycle, the patients were re-evaluated for tumor response by clinical examination and imaging studies according to the RECIST version 1.1 [15]. If the tumor had shrunk and was considered resectable, the patient was scheduled for surgery 3–4 weeks after completing induction chemotherapy. Surgical treatment consisted of either total laryngectomy with partial laryngectomy or total laryngectomy with total pharyngectomy and flap reconstruction (pectoralis major myocutaneous or radial forearm free flaps). The types of performed neck dissections were modified radical, radical, or extended radical. Chemoradiotherapy with weekly cisplatin (30 mg/m^2^) or carboplatin at an area under the curve of 1.5 combined with radiotherapy at the total dose of 60–70 Gy or radiotherapy alone at the dose of 66–70 Gy were recommended postoperatively.

Patients with unresectable tumors after induction chemotherapy were managed with concurrent chemoradiotherapy, radiotherapy, or supportive care according to their medical conditions. All patients were followed for a minimum of 12 months after treatment completion or until death. The institutional review board approved this study.

SPSS version 18.0 for Windows (IBM Corporation, Armonk, NY, USA) was used to analyze the patients’ characteristics. Variables were compared using Pearson’s chi-square or independent samples t-test, as appropriate. The Stata statistical software version 12.0 (Stata Corporation, Texas, USA) was used for survival analysis. Estimated survival function was calculated by using the Kaplan-Meier method and compared using the log-rank test. Logistic regression was used for univariate and multivariate analyses of factors affecting resectability. The Cox proportional hazards model was used for univariate and multivariate analyses of factors affecting survival function.

## Results

Thirty-two patients were included in the study; all had stage IVb diseases (T4bN3 = 12 patients, T4bN2 = 9 patients, and T3 N3 = 11 patients). The median follow-up time was 18 months (range, 6–35.5 months). According to our unresectability criteria, there were 9 patients with prevertebral fascia invasion, 17 with carotid artery encasement, 5 with both prevertebral fascia invasion and carotid artery encasement, and 1 with mediastinal structure involvement. The patient’s clinical characteristics are presented in Table 1. After 3 cycles of induction chemotherapy, none of the patients achieved complete tumor response. However, 21 patients had partial tumor response, 7 had stable disease, and 4 had progressive disease.

**Table 1:** Baseline Patient Characteristics

Characteristics	Resectable qroup *N* = 18 (%)	Unresectable group *N* = 14 (%)	*p*-value
Age, year			
Mean (SD)	59.5 (3.13)	62.3 (3.29)	0.053
Sex			
Male	14 (77.8)	10 (71.4)	0.681
Female	4 (22.2)	4 (28.6)	
Performance status			
0	12 (66.7)	7 (50)	0.341
1	6 (33.3)	7 (50)	
Smoking			
Yes	12 (66.7)	10 (71.4)	0.773
No	6 (33.3)	4 (28.6)	
Alcohol			
Yes	8 (44.4)	8 (57.1)	0.476
No	10 (55.6)	6 (42.9)	
Primary site			
Larynx	9 (50)	3 (21.4)	0.098
Hypopharynx	9 (50)	11 (78.6)	
TN stage			
T3 N3	6 (33.3)	5 (35.7)	0.888
T4bN2	5 (27.8)	4 (28.6)	
T4bN3	7 (38.9)	5 (35.7)	
Causes of unresectability			
Preveterbral fascia invasion	4 (22.2)	5 (35.7)	0.960
Carotid artery encasement	12 (66.7.)	5 (35.7)	
Preveterbral fascia invasion and carotid artery encasement	2 (11.1)	3 (21.4)	
Mediastinal structure involvement	0	1 (7.1)	
Regimen			
TPF	11 (61.1)	3 (21.4)	0.080
CP	3 (16.7)	5 (35.7)	
PF	4 (22.2)	6 (42.9)	
Differentiation			
Poor	7 (38.9)	7 (50)	0.641
Moderate	6 (33.3)	5 (35.7)	
Well	5 (27.8)	2 (14.3)	

TPF = docetaxel, cisplatin, and 5-fluorouracil, CP = carboplatin and paclitaxel, PF = cisplatin and 5-fluorouracil

Pathological examination of the surgical specimens revealed free, close, and positive margins in 1, 7, and 10 cases, respectively. Pathological extracapsular extension of the resected lymph node was observed in 9 cases.

Postoperative chemoradiotherapy was recommended in all surgical cases. Thirteen patients were able to complete the 6 cycles of chemotherapy, while 3 complete 1–3 cycles. Two patients had radiotherapy alone owing to a tumor-free surgical margin and no extracapsular extension of the dissected lymph node. Five patients with unresectable diseases were treated with concurrent chemoradiotherapy, 3 with radiotherapy, and 6 with supportive care. Pulmonary metastasis was noted in 3 patients during the follow-up period (12 months after surgery and adjuvant chemoradiotherapy in 1 patient, and 5 and 10 months after initiating supportive care in the other 2 patients, respectively).

### Resectability rate

Following induction chemotherapy, 18 patients with a partial response had sufficient tumor reduction and were considered resectable (i.e., the resectable group). The remaining 14 patients still had unresectable diseases (and comprised the unresectable group). The resectability rate was 56.3%.

### Factors predicting tumor resectability

Clinical variables including primary site, T stage, N stage, the cause of unresectability, chemotherapy regimen, and tumor differentiation were analyzed to determine tumor resectability after induction chemotherapy. On univariate analysis, laryngeal cancer and TPF regimen were the factors associated with tumor resectability (*p* = 0.048, and *p* = 0.006, respectively). Multivariate analysis showed that receiving a TPF regimen was the only predictive factor associated with producing sufficient tumor reduction; patients who underwent TPF had a tumor resectability rate of 78.6%, while those who underwent PF and CP regimens had resectability rates of 40% and 37.5%, respectively (*p* = 0.044). (Table 2)When considering the characteristics of resectable tumors, those with carotid artery encasement had the highest chance of undergoing surgical resection, with a rate of 70.6%. However, tumors with prevertebral fascia invasion alone, prevertebral fascia invasion plus carotid artery encasement, and mediastinal structure involvement had resection rates of 44.4%, 40%, and 0%, respectively; the differences were not significant (*p* = 0.088).

**Table 2:** Multivariate analysis of factors predicting tumor resectability after induction chemotherapy

Variable	Odds ratio	95% confidence interval	*p*-value
Chemotherapy regimen			
TPF vs. CP/PF	9.76	1.20–79.69	0.044

TPF = docetaxel, cisplatin, and 5-fluorouracil, CP = carboplatin and paclitaxel, PF = cisplatin and 5-fluorouracil

### Overall survival (OS)

The median OS of all patients was 16 months (range, 9.5–35.5 months). The median OS rates of the resectable and unresectable groups were 20.0 months (range, 16.0–35.5 months), and 9.5 months (range, 6.0–15.0 months), respectively (*p* = 0.008).

**Fig. 1:**
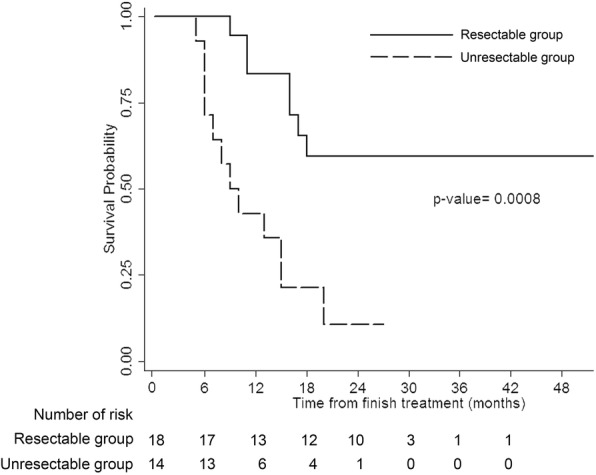
Kaplan-Meier curve of overall survival of patients in resectable group (solid line) and unresectable group (dashed line)

The estimated 2-year OS of all patients was 39.1% (95% confidence interval [CI], 22.1–55.7%). The estimated 2-year OS rates of the resectable and unresectable groups were 59.5% (95% CI, 33.2–78.3%), and 10.7% (95% CI, 1.1–35.4%), respectively (*p* = 0.0008) (Fig. 1).

### Disease-free survival (DFS)

The median DFS of all patients was 13.5 (range, 7.5–21.5 months). The median DFS of the resectable and unresectable groups were 20.0 months (range, 12.5–25.0 months), and 6.0 months (range, 5.0–11.0 months), respectively (*p* = 0.009).

**Fig. 2:**
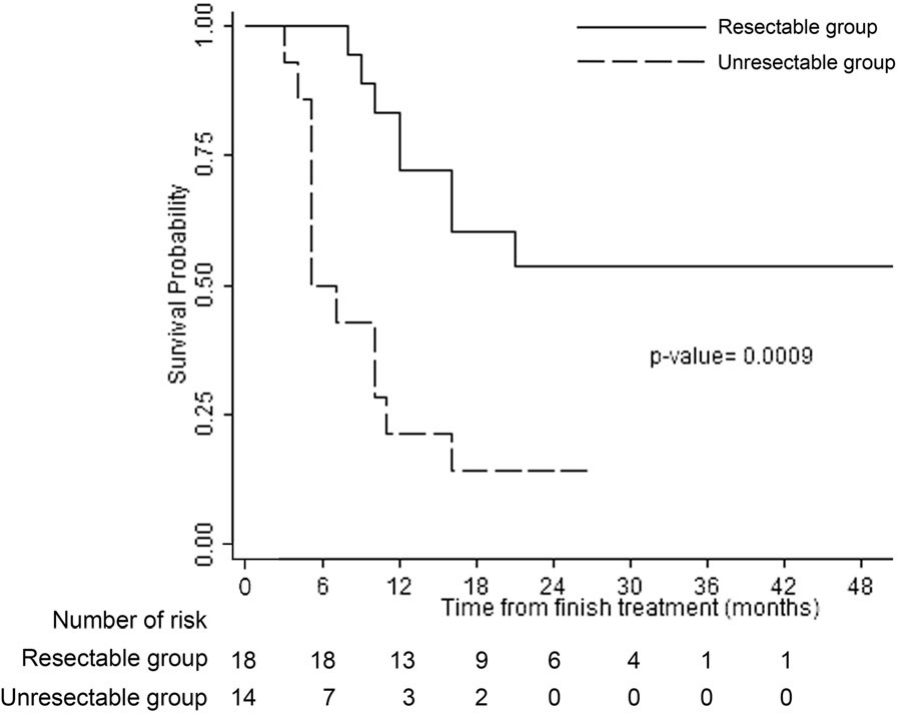
Kaplan-Meier curve of disease-free survival of patients of the resectable group (solid line) and unresectable group (dashed line)

The estimated 2-year DFS of all patients was 36% (95% CI, 19.6–52.7%). The estimated 2-year DFS of the resectable and unresectable groups were 53.5% (95% CI, 27.9–73.6%), and 14.3% (95% CI, 2.3–36.6%), respectively (*p* = 0.0009) (Fig. 2).

Locoregional recurrence occurred in 20 patients; 8 (44.4%) were in the resectable group and 12 (85.7%) in the unresectable group.

### Factors influencing OS and DFS

Clinical variables including age, sex, performance status, smoking, alcohol usage, primary site, T stage, N stage, the cause of unresectability, chemotherapy regimen, surgical resection, and tumor differentiation were analyzed to determine factors influencing OS and DFS rates in all patients. Univariate analysis revealed factors positively affecting OS and DFS rates were patients with performance status = 0 (*p* = 0.041, and *p* = 0.038, respectively), laryngeal cancer (*p* = 0.009, and *p* = 0.012, respectively), receiving the TPF regimen (*p* = 0.006, and p = 0.009, respectively), and surgical resection (*p* = 0.010, and *p* = 0.008, respectively).

However, on multivariate analysis, factors positively affecting the OS and DFS rates in all patients were surgical resection (*p* = 0.007 and 0.007, respectively), laryngeal primary (*p* = 0.009 and 0.005, respectively), and receiving the TPF regimen (*p* < 0.001 and = 0.006, respectively). (Table 3 and Table 4) No factor influencing OS and DFS were identified in the resectable group, while definitive chemoradiotherapy extended DFS in the unresectable group (*p* = 0.001).

**Table 3:** Multivariate analysis of factors positively affecting the overall survival rate

Variables	Hazard ratio	95% confidence interval	*p*-value
Primary site			
Larynx vs. Hypopharynx	0.14	0.03–0.60	0.009
Chemotherapy regimen			
TPF vs. CP/PF	0.08	0.01–0.45	< 0.001
Treatment			
Surgery vs. Non-surgery	0.26	0.09–0.69	0.007

TPF = docetaxel, cisplatin, and 5-fluorouracil, CP = carboplatin and paclitaxel, PF = cisplatin and 5-fluorouracil

**Table 4:** Multivariate analysis of factors positively affecting the disease-free survival rate

Variables	Hazard ratio	95% confidence interval	*p*-value
Primary site			
Larynx vs. Hypopharynx	0.12	0.03–0.53	0.005
Chemotherapy regimen			
TPF vs. CP/PF	0.09	0.02–0.51	0.006
Treatment			
Surgery vs. Non-surgery	0.27	0.10–0.69	0.007

TPF = docetaxel, cisplatin, and 5-fluorouracil, CP = carboplatin and paclitaxel, PF = cisplatin and 5-fluorouracil

## Discussion

For locally advanced resectable LHSCC, organ-preservation strategies using combined chemotherapy and radiotherapy as induction, concurrent, sequential, or alternating therapies have been studied in recent decades [16–18]. In more advanced LHSCC with cartilage invasion, extralaryngeal soft tissue invasion, or high-volume tumor, primary surgery with postoperative adjuvant therapy has remained the recommended therapy [16–19].

Induction chemotherapy in HNSCC has been aimed at reducing distant metastasis, inducing tumor shrinkage, and allowing for the assessment of tumor responsiveness in order to plan subsequent therapies [4]. Although induction chemotherapy followed by radiotherapy or chemoradiotherapy in locally advanced unresectable HNSCC (including LHSCC) showed an advantage for OS, the patients’ prognoses remained unsatisfactory [1, 10, 20]. Separately, induction chemotherapy has also been used to achieve surgical resection and improve prognosis in unresectable HNSCC [11, 12]. Patil et al. [11] investigated oral cavity cancer patients who had “technically unresectable tumors” because 1) the disease extended to the zygoma, 2) there was soft tissue involvement that reached the hyoid bone, 3) there was infratemporal fossa involvement, 4) oral tongue cancer extending to the vallecula was present, or 5) extensive soft tissue invasion impacting the achievement of a negative surgical margin was present. Approximately 43% of their patients experienced sufficient tumor shrinkage to allow surgery after induction chemotherapy. Schmaltz et al. [12] performed a study of patients with oral cavity cancer, oropharyngeal cancer, metastatic lymphadenopathy of unknown primary, and hypopharyngeal cancer; their criteria for unresectability were a high risk of incomplete surgical resection, advanced nodal status of N2b or more, thrombosis of the internal jugular vein, and major scalability. They achieved a resectability rate of 87.8% in their patients after induction chemotherapy.

The term ‘unresectable’ is difficult to define, as resectability has improved over time owing to advances in surgical and reconstruction techniques [11, 21]. The criteria for unresectability of the primary site or of the adenopathy generally include fixation to the spine or prevertebral muscles; or involvement of the skin, dura, base of skull, or brachial plexus [21]. We included patients with advanced unresectable laryngeal and hypopharyngeal cancers in our study. The criteria for unresectability were prevertebral fascia invasion, carotid artery encasement, and mediastinal structure involvement, which are classified as very advanced stage IVb (T4b and /or N3) by the AJCC Cancer Staging Manual, 7th edition. It revealed that hypopharyngeal cancers were detected more frequently to be unresectable in our patients because they are characterized by the extensive submucosal spread and high risk of nodal involvement which are usually more aggressive than laryngeal cancers [22]. To the best of our knowledge, there has been no study of the management of unresectable stage IVb LHSCC exclusively.

None of our patients achieved complete tumor response after 3 cycles of induction chemotherapy, but 18 patients with partial responses had sufficient tumor reduction for resection; their resectability rate was 56.3%. The only significant predictive factor for tumor response was receiving a TPF regimen. Among tumors that met our unresectability criteria, those with carotid artery encasement had the highest rate of resectability (70.6%) post-induction chemotherapy, but with no statistical significance.

Induction chemotherapy with the addition of docetaxel to cisplatin and 5-FU was previously shown to significantly improve OS and DFS in patients with unresectable HNSCC [1, 3, 11, 17]; this was also confirmed in our study. We also demonstrated that surgical resection had a positive impact on both OS and DFS, which is consistent with data from Patil et al. [11] and from Schmaltz et al. [12]. In primary site-specific analysis, patients with laryngeal cancer had longer survival than those with hypopharyngeal cancer.

In patients undergoing surgical resection, postoperative adjuvant therapy and favorable pathological response had previously been identified as factors that improved survival [11, 13]. However, none of these factors (including tumor stage, tumor differentiation, pathological surgical margin, pathological extracapsular extension of lymph node, or postoperative treatment) affected patient outcomes in our study. However, definitive chemoradiotherapy had a positive impact on DFS in the unresectable group.

The survival rates in our study appear to be lower than in patients who received sequential therapy for locally advanced larynx and hypopharynx cancer in the TAX 324 study [17]. However, this was expected since patients in our study had far more advanced disease stages. Therefore, the concept of surgical resection following induction chemotherapy, even in patients who initially had stage IVb disease, appears to be viable.

The limitations of our study include its retrospective nature, single-arm study, and small sample size. The chemotherapy regimen was decided based on the patient’s performance status, creatinine clearance and financial constraints. The TPF regimen was more likely to be administered in the resectable group, although there was no significant difference between the groups. However, this may cause selection bias and may affect the outcomes.

## Conclusion

In advanced unresectable stage IVb LHSCC, induction chemotherapy converted 56% of tumors to resectable status. Surgical resection following induction chemotherapy with TPF regimen appears to improve survival outcomes, especially in patients with laryngeal primary tumor sites. Concurrent chemoradiotherapy results in better DFS than either radiotherapy alone or supportive care in patients whose tumors remain unresectable after induction chemotherapy.

## Abbreviations

C: Carboplatin
CI: Confidence interval
DFS: Disease-free survival
F: 5-Fluorouracil
HNSCC: Head and neck squamous cell carcinoma
LHSCC: Laryngeal and hypopharyngeal squamous cell carcinoma
OS: Overall survival
P: Cisplatin
P: Paclitaxel
T: Docetaxel
TNM: Tumor/node/metastasis
